# A *Drosophila* model for mito-nuclear diseases generated by an incompatible interaction between tRNA and tRNA synthetase

**DOI:** 10.1242/dmm.019323

**Published:** 2015-08-01

**Authors:** Marissa A. Holmbeck, Julia R. Donner, Eugenia Villa-Cuesta, David M. Rand

**Affiliations:** 1 Department of Molecular Biology, Cell Biology, and Biochemistry, Brown University, Providence, RI 02912, USA; 2 Department of Ecology and Evolutionary Biology, Brown University, Providence, RI 02912, USA; 3 Department of Biology, Adelphi University, Garden City, NY 11530, USA

**Keywords:** Disease, Epistasis, Mitochondria

## Abstract

Communication between the mitochondrial and nuclear genomes is vital for cellular function. The assembly of mitochondrial enzyme complexes, which produce the majority of cellular energy, requires the coordinated expression and translation of both mitochondrially and nuclear-encoded proteins. The joint genetic architecture of this system complicates the basis of mitochondrial diseases, and mutations both in mitochondrial DNA (mtDNA)- and nuclear-encoded genes have been implicated in mitochondrial dysfunction. Previously, in a set of mitochondrial-nuclear introgression strains, we characterized a dual genome epistasis in which a naturally occurring mutation in the *Drosophila* *simulans simw^501^* mtDNA-encoded transfer RNA (tRNA) for tyrosine (tRNA^Tyr^) interacts with a mutation in the nuclear-encoded mitochondrially localized tyrosyl-tRNA synthetase from *Drosophila* *melanogaster*. Here, we show that the incompatible mitochondrial-nuclear combination results in locomotor defects, reduced mitochondrial respiratory capacity, decreased oxidative phosphorylation (OXPHOS) enzyme activity and severe alterations in mitochondrial morphology. Transgenic rescue strains containing nuclear variants of the tyrosyl-tRNA synthetase are sufficient to rescue many of the deleterious phenotypes identified when paired with the *simw^501^* mtDNA. However, the severity of this defective mito-nuclear interaction varies across traits and genetic backgrounds, suggesting that the impact of mitochondrial dysfunction might be tissue specific. Because mutations in mitochondrial tRNA^Tyr^ are associated with exercise intolerance in humans, this mitochondrial-nuclear introgression model in *Drosophila* provides a means to dissect the molecular basis of these, and other, mitochondrial diseases that are a consequence of the joint genetic architecture of mitochondrial function.

## Abstract (precis)

**Summary:** Interacting mutations in a mitochondrially encoded tRNA and nuclear-encoded tRNA synthetase result in a suite of pathologies, including altered locomotor capacity, mitochondrial function and morphology, and mitochondrial translation.

## INTRODUCTION

The endosymbiotic origin of mitochondria permitted gene transfer from the evolving mitochondrial genome to the nuclear genome. The result of this 2-billion-year association is that ‘mito-nuclear’ cross-talk is now vital to cellular function (Rand et al., 2004; Woodson and Chory, 2008). Mitochondria are semi-autonomous organelles that contain their own genome and are maternally inherited in most species. The mitochondrial genome encodes 13 of the polypeptide subunits involved in oxidative phosphorylation (OXPHOS), in addition to two ribosomal RNAs (rRNAs) and 22 transfer RNAs (tRNAs) required for mitochondrial protein translation (Rotig, 2011). Additional machinery required for mitochondrial protein synthesis [including aminoacyl-tRNA synthetases; initiation, elongation and termination factors; and ribosomal protein subunits) are encoded in the nucleus, imported into the mitochondria and integrated into the system for proper function (Woodson and Chory, 2008; Smits et al., 2010). Owing to the joint genetic architecture of this system, mitochondrial disorders can result from either mutations in the mitochondrial DNA (mtDNA) (Greaves et al., 2012) or in nuclear-encoded genes (nDNA) that function in the mitochondria (Rotig, 2011).

In higher organisms, the link between mutations in the mtDNA or nDNA and mitochondrial disease pathologies is often unclear. Combinations of genetic variants might result in synergistic interactions that alter phenotypes, making disease manifestation a complex problem (Klose, 1999). Additionally, environmental factors have the ability to modify phenotypes, resulting in intricate gene-gene-environment (G×G×E) interactions (Arnqvist et al., 2010; Zhu et al., 2014). The contribution of mtDNA mutations to disease traits is influenced by mito-nuclear interactions, and the pathogenic properties of a mtDNA mutation might depend on the nuclear background in which it is expressed (Dowling et al., 2008). This makes assessing the contribution of individual factors to the expression of complex disease traits difficult and, thus, examination of both mitochondrial and nuclear interacting factors is required to fully understand the dynamics that underlie mitochondrial disease expression.

In humans, over 500 point mutations and rearrangements in the mtDNA are associated with disease pathologies [Jacobs, 2003; Wong, 2007; Mitomap (http://mitomap.org/MITOMAP)]. Mitochondrial tRNA (mt-tRNA) genes compose less than 10% of the mitochondrial genome but are responsible for more than half of the identified mutations, implicating them as important disease targets. Mitochondrial tRNAs play a crucial role in the translation of mtDNA-encoded proteins in the mitochondrial matrix, where they interact with a nuclear-encoded partner, a mitochondrial aminoacyl-tRNA synthetase (mtAATS). The mtAATS is responsible for ‘charging’ the appropriate tRNA with the correct amino acid (Konovalova and Tyynismaa, 2013). Once charged, the tRNA brings the amino acid to the ribosomal complex for integration into a growing polypeptide chain based on complementary anticodon base-pairing. The interacting tRNA and synthetase are encoded in separate genomes, providing the opportunity for dual-genome epistatic interactions. Mutations in any component involved might result in translation and protein synthesis defects. In humans, mutations in either mt-tRNAs or their associated synthetases can result in a suite of pathologies, including respiratory-chain deficiencies (Kemp et al., 2011), exercise intolerance (Pulkes et al., 2000), anemia, encephalopathy and cardiomyopathy (Konovalova and Tyynismaa, 2013), among others. A simple model would predict that mutations in any mtAATS would result in general disruption of mitochondrial translation and similar pathologies but, interestingly, mtAATS mutations result in distinct clinical pathologies that are specific to the synthetase affected (Konovalova and Tyynismaa, 2013).

**TRANSLATIONAL IMPACT**

**Clinical issue**

Mitochondrial diseases often display patterns of incomplete penetrance, and the genotype-to-phenotype relationship is complex. The contribution of mitochondrial DNA (mtDNA) mutations to disease traits is influenced by both genetic and environmental (cellular and external) factors, and the pathogenic properties of a mtDNA mutation might depend on the nuclear background in which it is expressed. This makes it difficult to assess the contribution of individual factors to the expression of complex disease traits. Thus, the analysis of both mitochondrial and nuclear interacting factors is required to fully understand mitochondrial disease expression. *Drosophila* is an exceptionally useful animal model to investigate this because it allows the manipulation of the mitochondrial and nuclear genomes simultaneously. 

**Results**

The mtDNA from *Drosophila simulans* (*Dsim*) was introduced into *Drosophila melanogaster* (introgression) to generate strains that simultaneously express a naturally occurring mutation in the *Dsim* mtDNA and a mutation in the *Drosophila melanogaster* (*Dmel*) nuclear DNA*.* Both mutations affect genes that encode mitochondrial-function-related enzymes. This approach allowed the characterization of a dual-genome mitochondrial-nuclear incompatibility on a range of traits, providing a spectrum of the pathological phenotypes that result from the interacting mutations. The model is able to recapitulate a number of pathologies observed in human mitochondrial disease, including disrupted mitochondrial function, abnormal mitochondrial morphology and decreased exercise ability. A transgenic approach is used to rescue these deleterious phenotypes, identifying genetic interactions that could be manipulated for therapeutic purposes.

**Implications and future directions**

This study provides insights into the mitochondrial and nuclear genetic architecture that regulates cellular energy metabolism and influences the expression of complex mitochondrial disease traits. The research focuses on a specific example of a mtDNA mutation that displays different phenotypic effects based on the nuclear background in which it is expressed, and offers a plausible mechanistic explanation for the variable penetrance observed in human mitochondrial diseases. The results provide strong evidence that the combined analysis of mitochondrial and nuclear genotypes might be a better predictor of the physiological and disease consequences of mtDNA mutations. Additionally, these results suggest a paradigm for further characterization of mitochondrial diseases mechanisms, and for identifying potential therapeutic targets and strategies.

In order to dissect the role of mitochondrial-nuclear interactions in mitochondrial diseases, our lab previously developed a *Drosophila* model in which both genomes can be jointly manipulated (Rand et al., 2006; Montooth et al., 2010). mtDNA from *Drosophila simulans* (*Dsim*) was introduced across species boundaries to generate a series of novel strains that contain *Dsim* mtDNA in controlled *Drosophila melanogaster* (*Dmel*) nuclear backgrounds. A specific mito-nuclear combination displayed a strong interaction effect. When *simw^501^* (*Dsim*) mtDNA was paired with *OreR* (*Dmel*) nuclear chromosomes, a suite of abnormal phenotypes was observed, including: delayed development time, reduced fecundity and shortened scutellar bristles. These phenotypes appeared normal when other *Dsim* mtDNAs were placed on *OreR* chromosomes, or when the same *simw^501^* mtDNA was placed on an *Austria* (*Dmel*) nuclear background. These data implicated a locus in the *Austria* (*Aut*) nuclear genome that is able to rescue the performance defects unique to the (*simw^501^*); *OreR* (*mito; nuclear*) combination. Genetic mapping of *OreR* identified a mutation in the tyrosyl-mtAATS [aminoacyl-tRNA synthetase for tyrosine in the mitochondria: *Aats-tyr-m* (*Aatm*)], and sequencing of the complete *simw^501^* mtDNA revealed a potential interacting mutation in the mt-tRNA for tyrosine (tRNA^Tyr^). To confirm the source of this epistasis, a transgenic approach was used to generate rescue strains with genomic insertions of the alternative *OreR* and *Aut* alleles of *Aatm* (Meiklejohn et al., 2013). The *OreR* and *Aut Aatm* alleles differ by one nonsynonymous mutation in the *OreR* sequence that changes a highly conserved alanine to valine at amino acid position 275 in the Aatm peptide, and one synonymous site. An *Aatm* rescue allele was constructed using the complete *OreR Aatm* coding sequence but with a replacement of the single nonsynonymous single-nucleotide polymorphism (SNP) that restores the conserved alanine at position 275 of the Aatm protein. This rescue allele, referred to as *Aatm^Ore_V275A^*, allows us to test whether the A275V SNP is responsible for the incompatibility with the *simw^501^* mtDNA. Here, we use both the mito-nuclear introgression strains and transgenic rescue strains to develop a model for mitochondrial translation diseases.

In humans, mitochondrial diseases often display patterns of incomplete penetrance, and the genotype-to-phenotype relationship is complex (Zeviani and Di Donato, 2004; Schon et al., 2012; Riley et al., 2013). Thresholds for mtDNA mutations differ by organ and tissue type, and tissues with high OXPHOS demands (brain, heart, muscle, etc.) might be more sensitive to mtDNA mutations (DiMauro and Schon, 2003). In many instances the same genetic mutation varies in phenotypic effect, implicating the importance of environmental and genetic interacting factors (Jacobs, 2003). Here, we test whether the interacting mutations will differentially affect a variety of traits in multiple nuclear genetic contexts. The traits characterized range from being tightly associated biochemically to the interacting mutations and mitochondrial function, such as mitochondrial translation and OXPHOS enzyme activity, to multifactorial behavioral traits that differ in their energy demands, such as flight and climbing. Using this system, we test the hypothesis that the mito-nuclear genetic interaction exhibits uniform phenotypes versus varied pathologies that are trait specific. The goal of this study is to examine a more complete spectrum of the pathological capacity of interacting mitochondrial and nuclear mutations.

## RESULTS

Two sets of *Drosophila* strains were used in this study, referred to as ‘introgression strains’ and ‘rescue strains’. The first set contains four mito-nuclear introgression strains consisting of two different mtDNA genotypes, each of which is placed onto two different controlled, homozygous nuclear chromosomal backgrounds. The mito-nuclear introgression strains were generated by placing alternative *D. melanogaster OreR* mtDNA (*ore*) and *D. simulans simw^501^* mtDNA on two inbred wild-type *D. melanogaster* nuclear backgrounds, *OreR* and *AutW132* (supplementary material Table S1) (Montooth et al., 2010). The second set consists of transgenic *Aatm* rescue strains and matching sibling controls generated as described in Meiklejohn et al., 2013 (supplementary material Table S1 and Fig. S1). The rescue strains pair the *simw^501^* mtDNA with three alternative nuclear *Aatm* alleles: the defective *Aatm^Ore^*, the wild-type *Aatm^Aut^* or the transgenic rescue *Aatm^Ore_V275A^*. The transgenic *Aatm* strains allow us to test the phenotypic causality of the interacting mtDNA tRNA^Tyr^ and *Aatm* SNPs by isolating the nuclear interacting factor and placing the experimental alleles into a genetically controlled nuclear background to be tested in conjunction with the incompatible *simw^501^* mtDNA.

### Mito-nuclear incompatibility alters scutellar bristle length

**Fig. 1.**
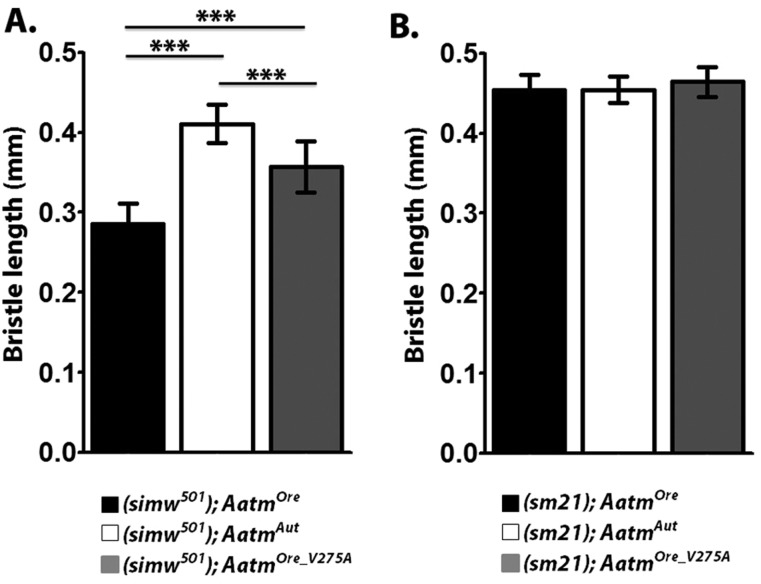
**Effects of the epistatic interaction on scutellar bristle length.** The transgenic rescue strains pair the *simw^501^* mtDNA with three alternative nuclear *Aatm* alleles: *Aatm^Ore^*, *Aatm^Aut^* or *Aatm^Ore_V275A^*. The sibling control strains are generated from the same cross (supplementary material Fig. S1). (A) Scutellar bristle length of transgenic rescue strains (mean±s.d., *n*=15, *F*_2,42_=79.48, ***P_ANOVA_<5e-15). The incompatible interaction shortens scutellar bristle length. (B) Bristle length measured in transgenic control sibling strains does not differ (mean±s.d., *n*=15, *F*_2,42_=0.285, *P_ANOVA_*=0.75).

Previously, we have shown that the mito-nuclear introgression strain (*simw^501^*); *OreR* (*mito; nuclear*) has shortened bristle length (Meiklejohn et al., 2013). To test whether the interacting mito-nuclear SNPs were responsible for the bristle phenotype, scutellar bristle length was measured in the set of transgenic *Aatm* rescue strains and sibling controls. When *simw^501^* mtDNA was paired with the *Aatm^Ore^* allele in a standardized and controlled nuclear background, bristle length was shorter compared with (*simw^501^*); *Aatm^Aut^* (*P_Tukey_*<0.0001) (Fig. 1A). When the transgenic rescue allele of *Aatm* was paired with the *simw^501^* mtDNA, this genotype, (*simw^501^*); *Aatm^Ore_V275A^*, displayed a partial rescue of bristle length, potentially due to additional modifiers in the nuclear background [*Aatm^Ore_V275A^* allele compared with both *Aatm^Ore^* and *Aatm^Aut^* (*P_Tukey_*<1.0e-5)]. Additionally, when the closely related *sm21* mtDNA from *D. simulans* was substituted in place of *simw^501^* mtDNA on the same set of transgenic nuclear backgrounds, no difference in bristle length was observed across the transgenic genotypes (Fig. 1B, *P_ANOVA_*>0.4). The *sm21* and *simw^501^* mtDNAs differ at six SNPs across their entire coding region: three synonymous sites (located in *ATPase6*, *ND1* and *ND5*), two changes at non-conserved sites in the large-subunit ribosomal RNA (lrRNA), and an SNP in the anticodon stem of the tRNA^Tyr^ gene; the latter is conserved across *Drosophila* species and is the putative functional defect in the *simw^501^* mtDNA (Meiklejohn et al., 2013). This demonstrates the specificity of interaction between the *simw^501^* mtDNA and *Aatm^Ore^* allele, and recapitulates our previous findings in the mito-nuclear introgression strains. The sibling control strains were generated from the same cross (supplementary material Fig. S1), and there were no differences in bristle length between the sibling controls in either the *simw^501^* or *sm21* transgenics (supplementary material Fig. S2A,B; *P_ANOVA_*>0.05 for both A and B).

### Flight ability is compromised in the epistatic genotype

**Fig. 2.**
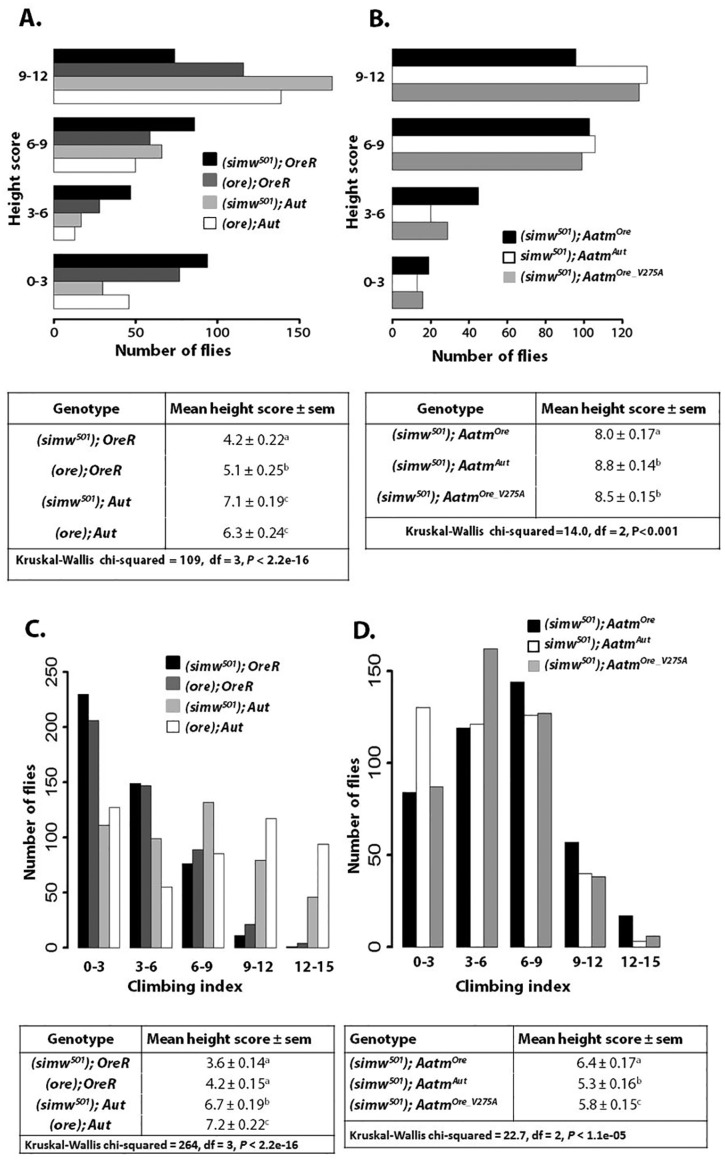
**The mito-nuclear incompatibility alters flight and climbing ability.** (A) Flight distributions of mito-nuclear introgression strains. The distribution of (*simw^501^*); *OreR* is downshifted and displays the lowest average flight score. (*simw^50^^1^*); *Aut* and (*ore*); *Aut* distributions do not differ significantly (*P_Wilcox_*=0.33). All other comparisons between genotypes are significantly different (pairwise contrasts in supplementary material Table S4). (B) Flight distributions of transgenic rescue strains. (*simw^501^*); *Aatm^Ore^* displays the lowest distribution of height scores. The distribution of (*simw^501^*); *Aatm^Ore^* differs significantly from (*simw^501^*); *Aatm^Aut^* and (*simw^501^*); *Aatm^Ore_V275A^* (pairwise contrasts in supplementary material Table S4). The distributions of (*simw^501^*); *Aatm^Aut^* and (*simw^501^*); *Aatm^Ore_V275A^* do not differ (*P_Wilcox_*>0.7). (C) Climbing profile of mito-nuclear introgression strains. (*simw^501^*); *OreR* and (*ore); OreR* distributions do not differ significantly (*P_Wilcox_*>0.1). All other comparisons between genotypes are significantly different (pairwise contrasts in supplementary material Table S4). (D) Climbing profile of transgenic rescue strains. The distribution of (*simw^501^*); *Aatm^Ore^* differs significantly from (*simw^501^*); *Aatm^Aut^* and (*simw^501^*); *Aatm^Ore_V275A^* (pairwise contrasts in supplementary material Table S4). The distributions of (*simw^501^*); *Aatm^Aut^* and (*simw^501^*); *Aatm^Ore_V275A^* are marginally different (*P_Wilcox_*=0.04). All *P-*values associated with Wilcoxon tests are Bonferroni-corrected to account for multiple testing. Different letters signify significant differences in pairwise contrasts.

In humans, diseases that compromise mitochondrial translation often result in exercise defects and early fatigue (Pulkes et al., 2000; Riley et al., 2010). *Drosophila* flight muscles are highly metabolically active and predominantly aerobic (Sacktor, 1976; Deak, 1977; Beenakkers et al., 1984), whereas climbing muscles favor anaerobic metabolism (glycolysis) as their energy source (Demontis et al., 2013). We performed flight and climbing (negative geotaxis) assays to measure two different aspects of locomotor performance as proxies for muscle function. Custom-designed flight chambers (modified from Benzer, 1973) were used to test whether the mito-nuclear incompatibility results in performance defects. A cylindrical column with a sticky coating contained height marks used to score where individual flies landed in the column; strong flyers land near the top and generate higher scores, whereas poor flyers fall lower on the column. In Fig. 2, the bars indicate the number of flies recorded at each height. Mito-nuclear introgression strains with *Aut* nuclear backgrounds displayed stronger flight ability than those with an *OreR* nuclear background (Fig. 2A). The *simw^501^* mtDNA further reduced flight capacity in the *OreR* nuclear background, but had no effect in the *Aut* background (supplementary material Table S4).

In the transgenic *Aatm* strains, flight ability displayed similar patterns to the mito-nuclear introgression strains. When the *Aatm^Aut^* or *Aatm^Ore_V275A^* alleles were paired with the *simw^501^* mtDNA, both strains displayed strong flight performance, and the distribution of the height scores were very similar (Fig. 2B, *P_Wilcox_*>0.7; supplementary material Table S4). (*simw^501^*); *Aatm^Ore^* displayed poor flight performance, with an overall lower distribution of height scores (supplementary material Table S4). Flight ability was not assessed in sibling control strains because the *Curly* wing marker is present on the second chromosome balancer and these flies do not fly (supplementary material Table S1).

### Climbing and flight performance have distinct mito-nuclear genetic bases

Climbing ability was measured to determine whether a distinct, complex locomotor trait that is less reliant on mitochondrial function would be influenced by the interacting mutations. Flies were placed into a climbing apparatus, and the vertical height that flies reached at a set time point was recorded to generate a climbing index. Over 300 flies per genotype were tested and the climbing index distributions are displayed in Fig. 2C,D. Similar to flight capacity, mito-nuclear introgression strains with *Aut* nuclear backgrounds were stronger climbers than *OreR* flies (Fig. 2C). The *simw^501^* mtDNA marginally reduced climbing ability in the *OreR* nuclear background, although the effect was not significant (*P_Wilcox_*>0.1, supplementary material Table S4). Unexpectedly, the *simw^501^* mtDNA significantly reduced climbing performance in the *Aut* nuclear background (Fig. 2C).

In the transgenic rescue strains, (*simw^501^*); *Aatm^Ore^* were the strongest climbers, whereas (*simw^501^*); *Aatm^Aut^* were the worst climbers (*P_Wilcox_*<4e-6) (Fig. 2D, supplementary material Table S4). (*simw^501^*); *Aatm^Ore_V275A^* displayed an intermediate distribution. There was no difference observed in the climbing ability of the transgenic control siblings (*P_Kruskal–Wallis_*=0.493) (supplementary material Fig. S3, Table S4). Interestingly, in the rescue strains, the flight and climbing performance of the genotypes was inverted. The strongest flyers were the poorest climbers, and the poorest flyers were the strongest climbers (Fig. 2B,D).

### Altered mitochondrial morphology from mito-nuclear interaction

**Fig. 3.**
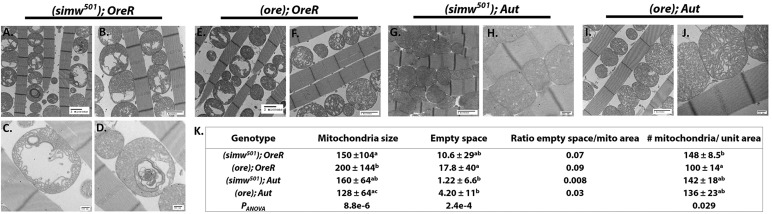
**Characterization of mitochondrial morphology in the mito-nuclear introgression strains.** TEMs of indirect flight muscle reveal structural abnormalities in (*simw^501^*); *OreR*. (A-D) (*simw^501^*); *OreR* display a loose cristae structure and matrix gaps. (*simw^501^*); *OreR* contained the most mitochondria per unit area, and several examples of swirled concentric cristae structures were observed (A,D). (E,F) (*ore*); *OreR* displayed some loose cristae structure and matrix gaps, and on average contained the largest mitochondria. (G-J) (*simw^501^*); *Aut* and (*ore*); *Aut* displayed tight cristae packing and healthy mitochondrial morphology. (K) Quantification of mitochondrial morphology parameters in mito-nuclear introgression strains. Approximately 100 mitochondria per genotype were analyzed to generate size data. Different superscript letters signify significant differences in pairwise comparisons (values for *P_Tukey_* pairwise contrasts are displayed in supplementary material Table S5). Mitochondria were counted in 50 μm×50 μm sections to generate density data. The average number of mitochondria per unit area of >3 sections are displayed. Scale bars: 2 μm (A,B,E-G,I); 500 nm (C,D,H,J).

Transmission electron microscopy was used to assess changes in mitochondrial morphology and structure associated with the mitochondrial phenotypes observed. Preparations from indirect flight muscles from the four mito-nuclear introgression genotypes are shown in Fig. 3. Similar to several of the other phenotypes characterized, the difference between the *OreR* and *Aut* nuclear backgrounds was pronounced, with the *simw^501^* mtDNA having a more significant impact in the *OreR* background. The cristae structure of (*simw^501^*); *OreR* mitochondria appeared loosely packed and displayed large gaps (Fig. 3A-D). Additionally, several instances of swirled cristae structure potentially due to cristae collapse were observed (Fig. 3C,D). (*simw^501^*); *OreR* contained the most mitochondria per unit area, potentially to compensate for mitochondrial defects (Fig. 3K). (*ore*); *OreR* also displayed large gaps in cristae structure, although no other instances of abnormal morphology were observed (Fig. 3E,F). The mitochondrial morphology of (*ore*); *Aut* and (*simw^501^*); *Aut* appeared healthier. The cristae were densely packed and fewer holes in cristae structure were observed. Quantification of mitochondrial ultrastructure measurements is displayed in the table in Fig. 3K (pairwise contrasts in supplementary material Table S5).

**Fig. 4.**
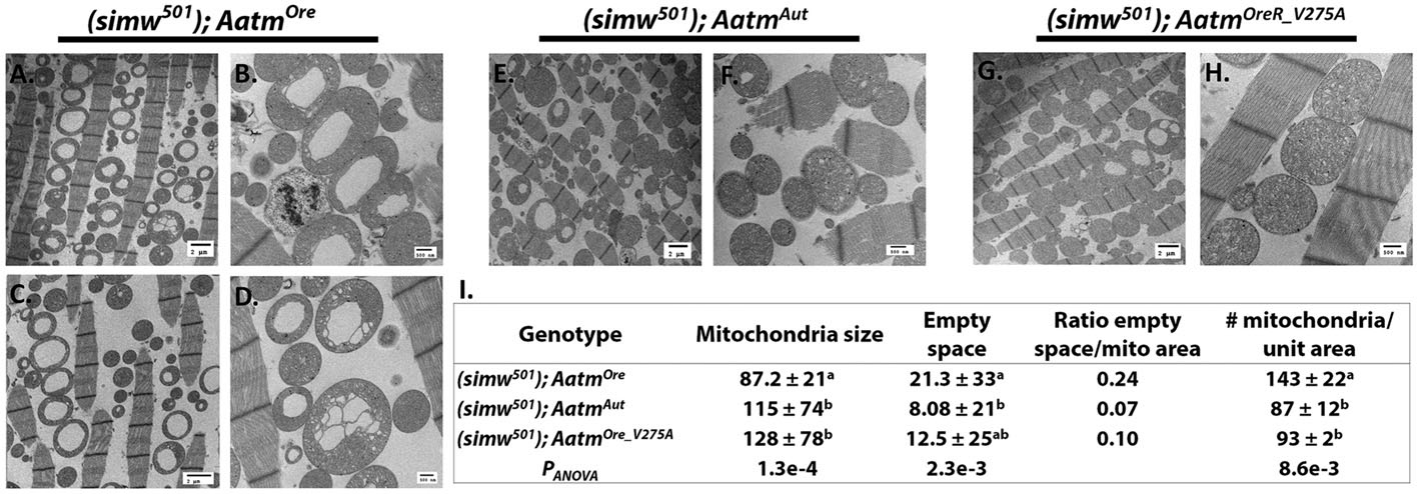
**Characterization of mitochondrial morphology in the transgenic rescue strains.** The incompatible mito-nuclear pair results in mitochondrial morphological defects. (A-D) Large matrix holes and unidentified inclusions were observed in (*simw^501^*); *Aatm^Ore^*. On average (*simw^501^*); *Aatm^Ore^* contains the smallest mitochondria, but the most per unit area. (E,F) (*simw^501^*); *Aatm^Aut^* displays some matrix holes, but maintains a relatively low ratio of empty matrix space to mitochondrial area. (G,H) The transgenic rescue genotype (*simw^501^*); *Aatm^Ore_V275A^* displays a healthy cristae structure and normal mitochondrial morphology. (I) Quantification of mitochondrial morphology parameters in transgenic rescue strains. Approximately 100 mitochondria per genotype were analyzed to generate size data. Different letters signify significant differences in pairwise comparisons (values for *P_Tukey_* pairwise contrasts are displayed in supplementary material Table S5). Mitochondria were counted in 50 μm×50 μm sections to generate density data. The average number of mitochondria per unit area of >3 sections are displayed. Scale bars: 2 μm (A,C,E,G); 500 nm (B,D,F,H).

In the transgenic *Aatm* strains, the defective genotype (*simw^501^*); *Aatm^Ore^* contained the most mitochondria per unit area (Fig. 4I and supplementary material Table S5), and displayed large gaps in cristae structure similar to (*simw^501^*); *OreR.* Several examples of severely abnormal morphology were observed (Fig. 4A-D), although these did seem to be distinct from the morphological defects seen in (*simw^501^*); *OreR* (Fig. 3C,D). The (*simw^501^*); *Aatm^Aut^* and the rescue genotype (*simw^501^*); *Aatm^Ore_V275A^* strains displayed fewer gaps in cristae structure (Fig. 4E-H). Mitochondrial size was smaller in the (*simw^501^*); *Aatm^Ore^* strain (average area of 87.2 μm), as compared to the (*simw^501^*); *Aatm^Aut^* and (*simw^501^*); *Aatm^Ore_V275A^* strains (average of 115 and 128 μm, respectively). A ratio of empty matrix space/overall mitochondrial area was calculated to quantify cristae structure gaps. This ratio was more than double in the (*simw^501^*); *Aatm^Ore^* strain as compared to the *Aatm^Aut^* or *Aatm^Ore_V275^* strains (ratio of 0.24 vs 0.07 and 0.10, respectively) (Fig. 4I).

### Mitochondrial respiration capacity is reduced in the (*simw^501^*); *OreR* combination

To test whether the identified mito-nuclear incompatibility alters overall mitochondrial oxidative capacity, respiration was measured *in vitro* in isolated mitochondrial preparations. In the mito-nuclear introgression strains, a nuclear background effect on mitochondrial respiratory capacity was observed. Introgression strains generated with *Aut* nuclear backgrounds displayed higher state III (ADP-dependent) respiration rates. No difference was seen in the state IV (ADP-independent) basal respiration rates (Table 1). An uncoupled rate was induced by addition of the chemical uncoupler FCCP to evaluate maximum OXPHOS capacity that is independent of membrane potential and ATP synthesis. (*simw^501^*); *OreR* displayed reduced state III and uncoupled rates, indicative of a lower overall maximum respiratory capacity (pairwise contrasts in supplementary material Table S6).

**Table 1.**
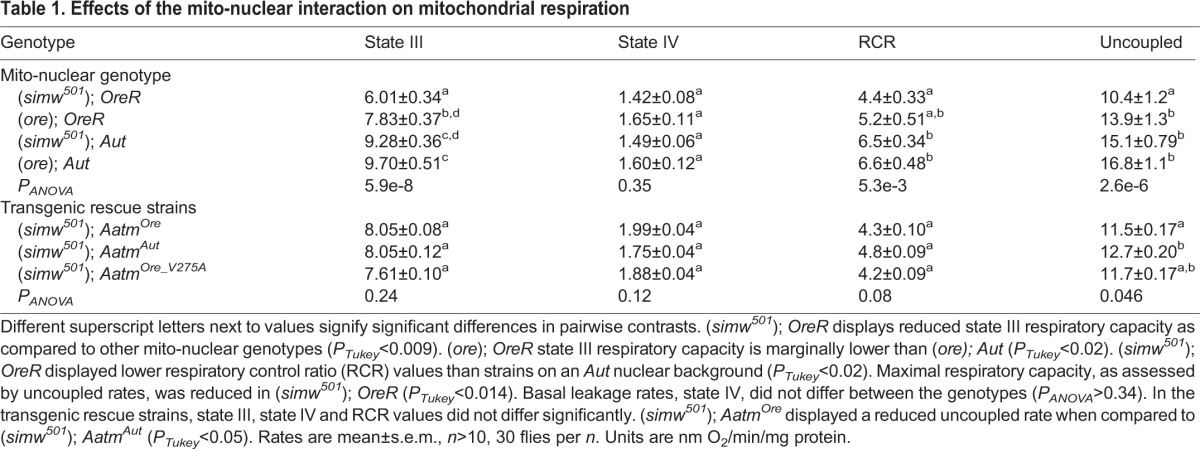
**Effects of the mito-nuclear interaction on mitochondrial respiration**

In the transgenic rescue strains, the defective genotype (*simw^501^*); *Aatm^Ore^* had reduced uncoupled rates compared with (*simw^501^*); *Aatm^Aut^*(*P_Tukey_*<0.05), indicating that the rate of maximal oxygen consumption is compromised, and the rescue genotype (*simw^501^*); *Aatm^Ore _V275A^* displayed an intermediate phenotype (Table 1, supplementary material Table S6). (*simw^501^*); *Aatm^Ore^* had comparable state III respiration rates to both (*simw^501^*); *Aatm^Aut^* and (*simw^501^*); *Aatm^Ore _V275A^* (*P_ANOVA_*=0.24, for *Aatm^Ore^*, *Aatm^Aut^* and *Aatm^Ore_V275A^* alleles). The transgenic control siblings displayed no difference in any of the respiratory measurements (*P_ANOVA_*>0.5, all parameters) (supplementary material Tables S2 and S6). The parameters measured are from functional and intact mitochondrial isolates, as all respiratory control ratio (RCR) values (state III O_2_ consumption rate/state IV O_2_ consumption rate) reported for all genotypes are >4 (Table 1), supporting the validity of the measured values (Schriner et al., 2009).

### Mito-nuclear incompatibility alters OXPHOS enzyme abundance and activity

**Fig. 5.**
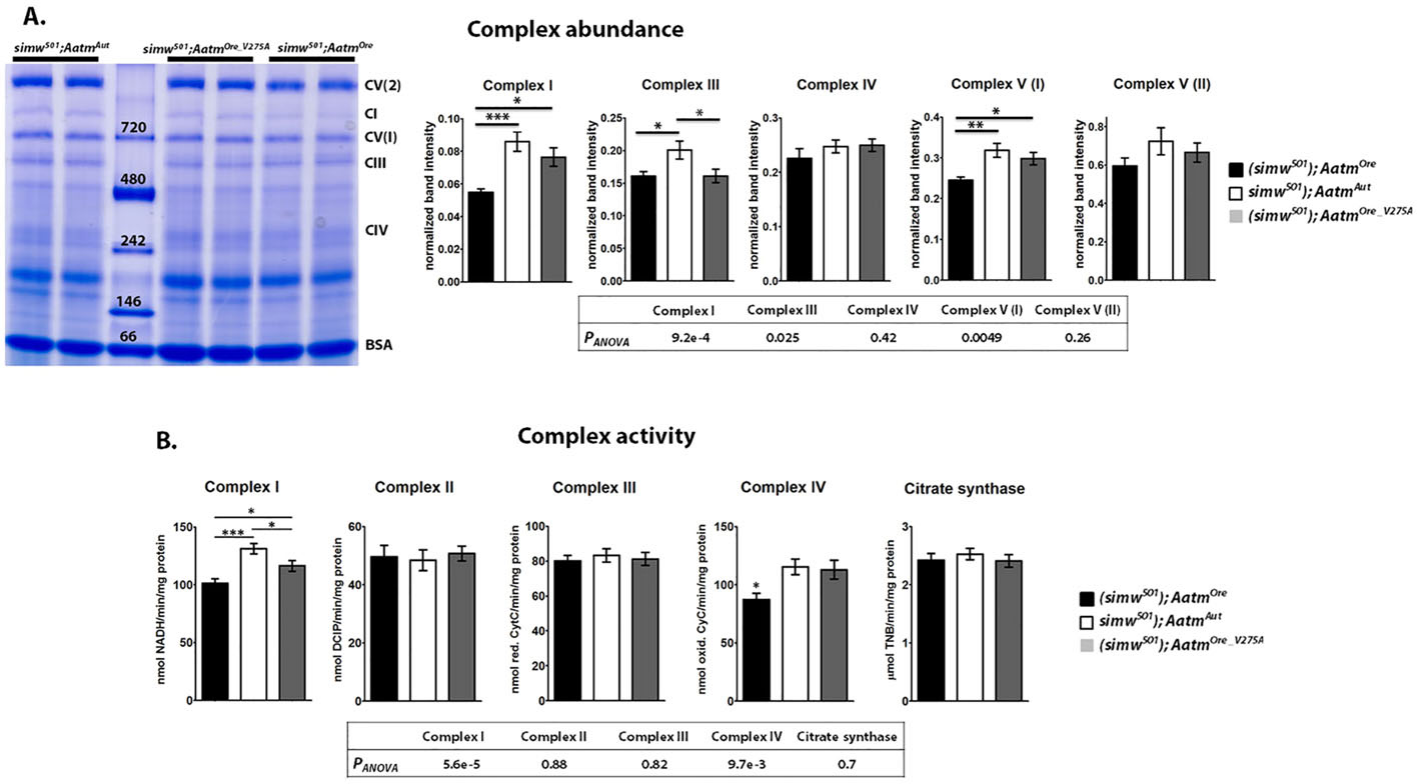
**OXPHOS enzyme abundance and activity is reduced in mitochondrially translated complexes containing the most tyrosines.** BN-PAGE analysis of mitochondrial protein isolates. (A) Example of BN-PAGE gel for transgenic rescue strains (note: full lanes moved from original gel locations for genotype consistency, no manipulations within lanes). Quantification of individual band intensity corresponding to Complex I, III and V relative to BSA. Across all complexes (*simw^501^*); *Aatm^Ore^* displayed reduced protein levels to varied degrees (individual contrasts in supplementary material Table S7). (B) Enzymatic activity of OXPHOS complexes. (*simw^501^*); *Aatm^Ore^* has significantly reduced activity of Complex I compared to (*simw^501^*); *Aatm^Aut^* and (*simw^501^*); *Aatm^Ore_V275A^* (*P_Tukey_*=3.18e-5 and *P_Tukey_*<0.05, respectively). The (*simw^501^*); *Aatm^Ore_V275A^* rescue strain has marginally lower Complex I activity compared with (*simw^501^*); *Aatm^Aut^* (*P_Tukey_*<0.05). Complex II and Complex III activity does not differ between the rescue strains (*P_ANOVA_*>0.8, each complex). Complex IV activity is reduced by the incompatibility in (*simw^501^*); *Aatm^Ore^* (*P_ANOVA_*<0.01). There is no difference in citrate synthase among the rescue genotypes (*P_ANOVA_*=0.7). The complexes with more mitochondrially encoded tyrosines display the most severe defect (Complexes I and IV). Enzymes that are completely nuclearly encoded (Complex II and citrate synthase) or those with few mitochondrially encoded tyrosines (Complex III) show little disruption of enzyme capacity. *P<0.05; **P<0.01; ***P<0.001.

To determine how the interacting mutations influence the relative abundance of assembled OXPHOS enzyme complexes, blue native polyacrylamide gels were performed. Amongst the transgenic rescue strains, the abundance of all complexes was reduced in (*simw^501^*); *Aatm^Ore^* compared with (*simw^501^*); *Aatm^Aut^* (Fig. 5A), with some variation across complexes. This effect was significant for Complexes I, III and V(I) (*P_Tukey_*<0.05, for all comparisons, supplementary material Table S7). The rescue genotype, (*simw^501^*); *Aatm^Ore_V275A^*, did not significantly differ from (*simw^501^*); *Aatm^Aut^* for Complex I, V(I) or V(II) abundance (*P_Tukey_*>0.4, for all comparisons, supplementary material Table S7). Across all complexes the rank order of the genotypes was maintained (Fig. 5A). In the transgenic sibling controls there were no differences detected in the abundance of any of the mitochondrial complexes (supplementary material Fig. S4, *P_ANOVA_*>0.6, for all comparisons in all complexes, supplementary material Table S7).

To assess functionality of OXPHOS complexes, enzyme activity was measured *in vitro*. Previously in the introgression strains we found that complexes jointly encoded by both mtDNA and nDNA (Complex I, Complex III and Complex IV) were compromised in (*simw^501^*); *OreR*, whereas Complex II and citrate synthase, both of which are completely nuclearly encoded, showed no difference among the introgression genotypes in enzyme activity (Meiklejohn et al., 2013).

To determine whether the *simw^501^*–*Aatm* interaction was responsible for these phenotypes, complex activity was evaluated in the transgenic rescue strains. (*simw^501^*); *Aatm^Ore^* displayed compromised Complex I and IV activity (Complex I *P_ANOVA_*<6e-5, and Complex IV *P_ANOVA_*<0.01) (Fig. 5B, supplementary material Table S7). (*simw^501^*); *Aatm^Ore_V275A^* was able to partially or fully rescue these effects. The severity of the defect corresponds in rank order with the number of mitochondrially encoded tyrosines present in each complex (supplementary material Table S3). Complex I was more severely compromised than Complex IV; this is consistent with a model in which the number of tyrosines that are mitochondrially translated will determine the severity of the defect. In the transgenic sibling controls there was no difference in complex activity (*P_ANOVA_*>0.3, each complex). Citrate synthase activity was marginally reduced in (*simw^501^*); *Aatm^Aut^* Sib (*P_ANOVA_*>0.02) (supplementary material Fig. S4, Table S7).

### Mito-nuclear incompatibility alters translation initiation rates in the mitochondria

**Fig. 6.**
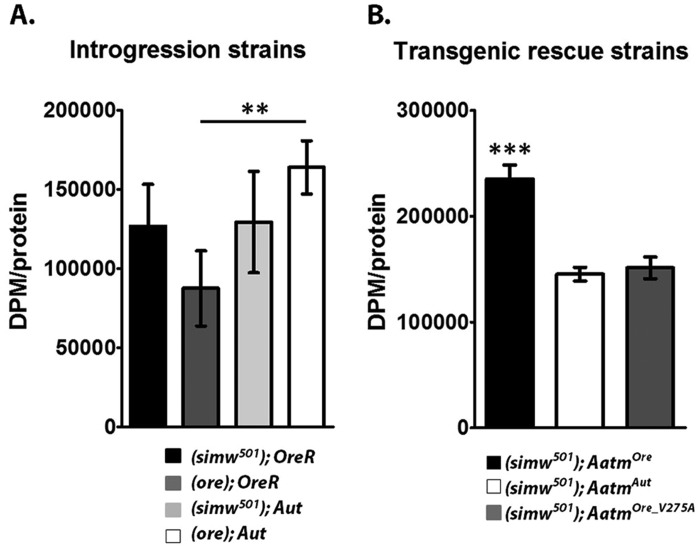
**Mitochondrial translation is upregulated by the mito-nuclear incompatibility.** Quantification of *de novo* translation as assessed by ^35^S-methionine incorporation into newly synthesized proteins, normalized to total mitochondrial protein. (A) ^35^S-methionine incorporation in introgression strains. (*simw^501^*); *OreR* displays a mild increase compared to (*ore*); *OreR*, and no significant difference is seen between (*simw^501^*); *OreR* compared to either (*simw^501^*); *Aut* or (*ore*); *Aut*. (B) ^35^S-methionine incorporation is significantly increased in (*simw^501^*); *Aatm^Ore^* compared to the other transgenic rescue strains (*P_Tukey_*<4e-6 for both comparisons). DPM, disintegrations per minute. **P<0.01; ***P<0.001.

*De novo* translation initiation was measured via ^35^S-methionine incorporation to assess how the interacting mutations, which putatively influence tRNA^Tyr^ charging, alter protein synthesis rates. Adult flies of each genotype were placed on standard media supplemented with 100 µCi/ml ^35^S-methionine for 48 h. Mitochondrial fractions were prepared, and ^35^S-methionine incorporation was found to differ amongst the mito-nuclear introgression strains (*P_ANOVA_*<0.005), and strains with *Aut* nuclear backgrounds displayed higher rates of mitochondrial translation initiation (Fig. 6A). In the *OreR* nuclear background the *simw^501^* mtDNA increased mitochondrial translation initiation, although this effect did not reach significance (supplementary material Table S8, *P_Tukey_*=0.12). This result was confirmed in an independently performed experiment (*P_t-test_*<0.02, supplementary material Fig. S6, data not combined as ^35^S-methionine batch differed and *Aut* introgression strains were not run). Translation initiation rates in (*simw^501^*); *OreR* did not differ significantly from either of the *Aut* introgression strains (Fig. 6A, supplementary material Table S8).

In the transgenic rescue strains a similar pattern was observed, and translation initiation rates were increased in (*simw^501^*); *Aatm^Ore^* compared to either (*simw^501^*); *Aatm^Aut^* or (*simw^501^*); *Aatm^Ore_V275A^* (Fig. 6B, *P_Tukey_*<4e-6 for both comparisons). Additionally, no difference was observed between (*simw^501^*); *Aatm^Aut^* and (*simw^501^*); *Aatm^Ore_V275A^* (Fig. 6B, *P_Tukey_*=0.9). In the transgenic sibling controls ^35^S-methionine incorporation was also marginally increased in (*simw^501^*); *Aatm^Ore^* Sib compared to (*simw^501^*); *Aatm^Aut^* Sib (supplementary material Fig. S5, *P_ANOVA_*=0.013, supplementary material Table S8).

## DISCUSSION

Here we characterize a broad range of phenotypes that are affected by a mitochondrial-nuclear incompatibility. In a previous study, we showed that a mutation in the mtDNA-encoded tRNA^Tyr^ interacted negatively with a mutation in the nuclear-encoded aminoacyl-tRNA synthetase for tyrosine in the mitochondria, *Aats-tyr-m* (*Aatm*) gene. Transgenic rescue strains confirmed that the nuclear mutation was causally associated with a developmental delay in this system (Meiklejohn et al., 2013). The present study builds on this model and here we analyze the pathological outcomes of the interacting mutations starting with whole organismal phenotypes and working down to the level of mitochondrial function and translation. By comparing the specific mito-nuclear interaction in two sets of genetic backgrounds (‘introgression strains’ and ‘transgenic rescue strains’), we have shown that the incompatible mito-nuclear ‘disease’ genotype has distinct phenotypic consequences across different traits. The tissue-specific or variable penetrance of mitochondrial dysfunction is a common condition in human mitochondrial diseases, and this *Drosophila* model offers a promising system for dissecting this complexity.

### The mito-nuclear incompatibility affects organismal performance and morphological phenotypes

A mutation identified in the human tyrosyl-tRNA synthetase gene, *YARS2*, primarily affects muscles and results in fatigue (Konovalova and Tyynismaa, 2013). We examined locomotor traits in our system and found that the *simw^501^* and *Aatm^Ore^* mutations impact flight and climbing ability differentially, and these effects were nuclear background dependent. During insect flight a number of enzymes involved in aerobic metabolism have been found to function at near maximum capacity (Suarez et al., 1996), and flight muscles might account for >90% of organismal oxygen consumed (Suarez, 2000). The interacting mutations in (*simw^501^*); *OreR* likely contribute to subpar flight performance due to decreased aerobic metabolic capacity, supported by the reduced mitochondrial respiratory capacity and enzyme activity described below. Additionally, the flight defect observed in the defective genotype is consistent between the introgression strains and transgenic rescue strains.

Analysis of climbing ability did not reveal a consistent defect between the introgression strains and transgenic rescue strains when the *simw^501^* and *Aatm^Ore^* mutations co-occur. Flight and climbing are separable behaviors that utilize morphologically distinct muscle groups in *Drosophila* (Peckham et al., 1990; Rhodenizer et al., 2008; Augustin and Partridge, 2009). Flight muscles contain abundant large mitochondria (30-40% of fiber volume) (Shafiq, 1963; figure 2 in O'Donnell et al., 1989), whereas leg muscles required for jumping and climbing [tergal depressor of the trochanter (TDT) and tergotrochanteral depressor muscle (TTM)] contain small and infrequently distributed mitochondria (figure 4 in O'Donnell et al., 1989). The metabolic profiles of these muscles also differ in insects; indirect flight muscles display a very low LDH/GAPDH ratio, and high TCA cycle activity (LDH/GAPDH, see figure 1 and table 2 in Beenakkers et al., 1984; SDH and IDH, see figure 1 in Deak, 1977), but TDT and TTM do not. The signature of enzymatic activity observed in indirect flight muscles is indicative of exclusive substrate processing in the mitochondria.

In mitochondrial disease states these muscle types are also differentially affected: knockdown of *Drosophila* proteins that play a role in mitochondria quality control (*PINK1* and *parkin*) results in severe disruption of muscle fiber arrangement in the indirect flight muscles, whereas leg muscles are only mildly affected (Pesah et al., 2004; Yang et al., 2006). Additionally, *Parkin* mutants completely lack the capacity to fly, whereas jumping ability is partially maintained (Greene et al., 2003; Pesah et al., 2004). Thus, different energy demands of muscle types likely contribute to the contrast observed in the transgenic rescue strains, where (*simw^501^*); *Aatm^Ore^* has decreased flight performance but increased climbing ability. Based on muscle type differences described above it is predicted that flight but not climbing behavior will be more tightly linked to the interacting tRNA-synthase mutations. Additionally, we observed that flight capacity is higher across all transgenic rescue strains compared to the introgression strains, whereas climbing ability falls within a similar range across the groups. The introgression strains have inbred nuclear backgrounds whereas the transgenic rescue strains are F1 hybrids, potentially masking any deleterious recessive alleles in the nuclear genome and resulting in enhanced flight ability. This difference in genetic background between the introgression and the transgenic rescue strains might account for some of the differences in rescue effects in these two sets of strains.

A third organismal trait found to be influenced by the *simw^501^*–*Aatm* interaction was bristle length. We previously showed that (*simw^501^*); *OreR* results in a short bristle phenotype (Meiklejohn et al., 2013), and these results are recapitulated in the (*simw^501^*); *Aatm^Ore^* strain. We find that the *Aatm^Ore_V275A^* allele is only able to partially rescue this effect, indicating that the *Ore* A275V SNP is the main effect, but subtle background effects could be modifiers. Additionally, when a different mtDNA is placed on this background, (*sm21*); *Aatm^Ore^*, bristle length is completely restored, demonstrating that both mutations are required for the complete bristle defect. The bristle phenotype is similar to those previously identified in *Drosophila Minute* mutants in which cytosolic ribosomal components are disrupted. Translational defects result in reduced growth because protein synthesis cannot keep up with cellular demands (Lambertsson, 1998). A mutation in the mitochondrial import machinery, Tim50, results in a similar bristle defect, reduced cell proliferation and growth defects. Defective mitochondrial protein import likely results in suboptimal mitochondrial performance and the inability to produce sufficient energy, resulting in these growth defects (Sugiyama et al., 2007). Thus, defects in protein synthesis are a common component in other genetic models that result in short bristles, lending support for the model of defective mitochondrial translation presented here.

At the subcellular level, the *simw^501^*–*Aatm* interaction revealed dramatic effects on mitochondria morphology, including large gaps in the mitochondrial matrix and a collapsed cristae structure similar to those seen in human mitochondrial diseases (Carta et al., 2005; Cogliati et al., 2013). The defects observed in (*simw^501^*); *OreR* and (*simw^501^*); *Aatm^Ore^* appear morphologically distinct (Figs 3 and 4), and the hollow matrix is more severe and smooth in (*simw^501^*); *Aatm^Ore^*. Both of the defective genotypes in the introgression and transgenic rescue strains displayed an increase in mitochondrial number, which could be indicative of a compensatory mechanism to maintain energy production levels (discussed more below).

In *Drosophila*, disruption of proteins that play a role in mitochondria quality control, *PINK1* and *parkin*, results in similar mitochondrial morphological defects, including swollen mitochondria, fragmented cristae structure and matrix degeneration (Park et al., 2006; Saini et al., 2011; Klein et al., 2014). In the case of the *simw^501^* mtDNA and *Aatm^Ore^* interaction, it seems that mutations in both genomes have the ability to affect mitochondrial morphology. The defective mitochondrial complexes might be influencing structure formation, and previous studies have noted that specific complex dimerization can regulate membrane curvature (Dudkina et al., 2005; Daum et al., 2013). Additionally, a stoichiometric imbalance of mitochondrial and imported nuclear-encoded proteins might also influence structural properties (Houtkooper et al., 2013). Thus, our genetic model might provide the means to dissect mtDNA and nDNA contributors to mitochondrial morphology.

### Alteration of mitochondrial function by the mito-nuclear incompatibility

A number of mitochondrial functional parameters were measured in live respiring mitochondria as well as frozen isolated samples. Integrated OXPHOS measurements of state III and uncoupled respiration rates revealed a strong nuclear background effect in the introgression strains (*OreR* nuclear background displayed lower rates than *Aut*), indicating that additional nuclear factors are influencing mitochondrial respiratory performance in conjunction with the identified tRNA-synthetase interaction. In the transgenic strains, uncoupled respiration was reduced in (*simw^501^*); *Aatm^Ore^* compared to (*simw^501^*); *Aatm^Aut^*, supporting the main effect of the tRNA-synthetase interaction, and suggesting that the mito-nuclear incompatibility results in decreased OXPHOS and O_2_ consumption, potentially contributing to some of the locomotor phenotypes characterized.

When analyzing individual OXPHOS enzyme complexes, we previously showed that all complexes jointly encoded by mtDNA and nDNA were compromised in (*simw^501^*); *OreR* (Meiklejohn et al., 2013). Upon controlled dissection of the interaction with the transgenic (*simw^501^*); *Aatm^Ore^* strain we observed a pattern of disruption that corresponds with the number of mitochondrially translated tyrosines present in each enzyme complex. The most severely affected complex, Complex I, contains the most tyrosines (98), followed by Complex IV with 39 tyrosines. The (*simw^501^*); *Aatm^Ore_V275A^* strain was able to partially rescue complex I activity and fully rescue complex IV activity. This implicates the tRNA-synthetase interaction as a main effect, but additional factors are likely to be involved. Complex III contains only one mtDNA-encoded subunit and 19 tyrosines. A defect in Complex III was not observed in the transgenic strains, potentially due to a lack of sensitivity in the assay, in addition to a predicted milder effect because fewer mitochondrially encoded tyrosines need to be incorporated into this complex. In the *simw^501^*–*Aatm* interaction we find that the absolute number of tyrosines in each complex is a better predictor of the severity of the defect than the proportion of tyrosine present (which is relatively consistent across complexes). This supports a model in which each tyrosine that must be incorporated into the protein complex can disrupt mitochondrial translation. In humans, similar defects in OXPHOS are observed when mitochondrial protein synthesis is compromised (Jacobs, 2003), and specific tRNA mutations result in varied biochemical defects, potentially due to the amino acid composition of each complex.

### Compensatory mitochondrial biogenesis and retrograde signaling

Based on the physical location of the interacting mutations in our model, it is predicted that aminoacylation of the tRNA^Tyr^ is compromised (Moreno-Loshuertos et al., 2011; Meiklejohn et al., 2013). In turn, this should impact translation rates and any functions that depend on mtDNA-encoded proteins. Our experiments on translation initiation reveal that genotypes harboring the incompatible mutations show elevated levels of ^35^S-methionine incorporation, whereas no such effect was observed when the *Aatm^Ore_V275A^* rescue allele was paired with the *simw^501^* mtDNA. These results are consistent with the hypothesis that the defective mito-nuclear genotypes are mounting a compensatory response by stimulating additional translation. These results agree with additional evidence that mtDNA copy number is increased in (*simw^501^*); *OreR* compared to the other mito-nuclear genotypes (Zhu et al., 2014). Upregulation of mtDNA has been proposed as a compensatory mechanism of mt-tRNA mutations (Moreno-Loshuertos et al., 2011), and has been observed in humans with mitochondrial diseases (Yen et al., 2002). In further support of compensatory mitochondrial biogenesis, we observed increased mitochondrial numbers in both the introgression and transgenic rescue strains harboring the *simw^501^* and *Aatm^Ore^* mutations (Figs 3 and 4).

The increase in mtDNA copy number, translation initiation and mitochondrial number indicate that the cell is able to sense disruption of mitochondrial activity and respond in an attempt to maintain function. Mitochondrial replication, transcription and translation require the import of nuclear proteins (Smits et al., 2010). The proposed functional consequences of the interacting mutations lies within the mitochondrion, suggesting that a mitochondrial retrograde signal mediates a nuclear transcriptional response to generate the necessary proteins required for increased mtDNA replication, translation and structural components. Retrograde signaling pathways result in the activation of nuclear transcription factors, and one relevant example might be the transcriptional coactivator PGC1α (peroxisome proliferator-activated receptor γ, coactivator 1 α), which is a master regulator of mitochondrial biogenesis in mammals and is induced by mitochondrial dysfunction (Butow and Avadhani, 2004; Chae et al., 2013). Here we observed decreased OXPHOS complex abundance and activity in the transgenic rescue strain carrying both the *simw^501^* and *Aatm^Ore^* mutations, indicating that the compensatory upregulation of mitochondrial translation initiation cannot fully offset the effects of these mutations. Additionally, without this putative compensatory response, the pathological outcome of the interacting mutations might be more severe.

### Nuclear modifiers of the epistatic interaction

In general, the ‘introgression strains’ and ‘transgenic rescue strains’ display similar phenotypic outcomes for the *simw^501^*–*Aatm* interaction. However, there are traits for which these strains show distinct phenotypes, suggesting that nuclear modifiers of the epistatic interaction exist. The mito-nuclear introgression strains were built on two separate sets of nuclear chromosomes, *OreR* and *Aut*. Nuclear chromosomal effects, in which *Aut* strains outperform *OreR* strains, were apparent in a number of phenotypes characterized, including mitochondrial respiration (Table 1: state III respiration), flight ability (Fig. 2A) and climbing ability (Fig. 2C). In these whole-genome introgression strains there are likely a large number of factors that differ between the *OreR* and *Aut* genomes that impact mitochondrial function, some of which might directly influence the effects of the *simw^501^*–*Aatm* interaction.

In order to control for these chromosomal effects and to test the *simw^501^*–*Aatm* interaction in a common nuclear background, transgenic strains were developed (Meiklejohn et al., 2013). We find that the *simw^501^*–*Aatm* interaction impacts a number of traits, and the phenotypes are influenced by nuclear background (Fig. 2A vs 2B: flight; Fig. 2C vs 2D: climbing; Table 1: mitochondrial state III respiration). Additionally, the transgenic rescue strains reveal that the *Ore* A275V SNP is the main effect, but subtle background effects could be modifiers that result in partial rescue of the primary defect. When characterizing the transgenic rescue strains in several of the phenotypes measured, the *Aatm^Ore_V275A^* allele displayed a partial rescue when compared to the *Aatm^Aut^* allele (Fig. 1: bristle length; Fig. 5: complex I activity). In other traits the *Aatm^Ore_V275A^* allele was able to fully rescue the defective phenotype and was indistinguishable from *Aatm^Aut^* (Fig. 2B: flight; Fig. 5: complex III and IV activity). These different traits seem to have additive or dominant effects in the *simw^501^*–*Aatm* interaction. Additionally, there was no clear dosage effect in the transgenic sibling controls. In most parameters measured the transgenic sibling control strains displayed similar performance (supplementary material Table S2, Figs S3 and S4), indicating that a single compatible *Aatm* allele is sufficient to rescue the deleterious phenotypes.

The mitochondrial defects displayed variable penetrance in the *simw^501^*–*Aatm* interaction, implying that specific traits might be differentially susceptible to the main effect of the interaction. Traits that are strongly influenced by mitochondrial translation and function (OXPHOS complex activity, Fig. 5) display distinct patterns of disruption corresponding to the *simw^501^*–*Aatm* interaction, whereas multifactorial traits with less direct biochemical dependence on mitochondria (climbing, Fig. 2) show mild or no disruption. This highlights how the pathogenic properties of mtDNA mutations can be altered by the nuclear background in which they are expressed, and these effects mirror the complexity observed in human mitochondrial diseases. Our model could provide an effective means to dissect this complexity and can be used to screen for additional nuclear factors that modify the *simw^501^*–*Aatm* interaction.

### Human diseases of mitochondrial translation

In humans, separate pathogenic mutations have been identified in both the mitochondrial tRNA^Tyr^ and nuclear tyrosyl-tRNA synthetase genes. The mutations in the mitochondrially localized tyrosyl-tRNA synthetase gene, *YARS2*, result in mitochondrial myopathy, lactic acidosis and sideroblastic anemia (MLASA syndrome) (Konovalova and Tyynismaa, 2013). Similar to the phenotypes seen in our flies, affected individuals display exercise intolerance and decreased electron transport chain (ETC) complex activity (Riley et al., 2013; Shahni et al., 2013). Interestingly, two patients homozygous for the same mutation (p.Phe52Leu) varied in the severity of the disease pathologies presented; one being mildly affected whereas the other was severely affected (Riley et al., 2013). These patients had different mtDNA haplogroups and nuclear genetic material, possibly contributing to the observed phenotypic variability. In a separate study a pathogenic mutation associated with exercise intolerance was identified in the mitochondrial tRNA^Tyr^ gene (Pulkes et al., 2000). The specific phenotypes and similarities observed between humans with mitochondrial tRNA and synthetase mutations and the flies described here demonstrates the effectiveness of our *Drosophila* model, and exemplify the complexity of mitochondrial diseases.

Recent studies have shown that overexpression of specific mtAATSs is able to partially rescue molecular defects resulting from pathogenic mt-tRNA mutations in cell lines (Rorbach et al., 2008; Li and Guan, 2010; Perli et al., 2014; Tran Hornig-Do et al., 2014). In one example, the mt-tRNA^Val^ mutation (m.1624C>T) resulted in destabilization of the uncharged tRNA, and overexpression of the nuclear synthetase, VARS2, was able to increase charging of the mutant tRNA and rescue molecular phenotypes (Rorbach et al., 2008). Our results demonstrate that expression of a compatible *Aatm* allele is able to partially or fully rescue a series of deleterious phenotypes when paired with *simw^501^* mtDNA at the level of a whole organism. Several additional studies demonstrate the effectiveness of *Drosophila* models in dissecting mitochondrial diseases (Toivonen et al., 2001; Bayat et al., 2012; Vartiainen et al., 2014). Currently there are no effective treatments for mitochondrial tRNA diseases in humans. Our study and those described above suggest that expression and import of nuclear synthetases might provide a novel therapy for mitochondrial tRNA diseases. The recent approval of mitochondrial replacement therapies is one promising approach to treating these diseases, but the mito-nuclear interactions resulting from such ‘three-parent babies’ raise the possibility of unintended interactions between mtDNA and nDNA SNPs that could generate novel pathologies (Reinhardt et al., 2013). Additional studies examining the complexity of mito-nuclear genetic interactions are warranted as these treatments move into actual clinical practice.

## MATERIALS AND METHODS

### *Drosophila* stocks and maintenance

The four mito-nuclear genotypes used in this study are a subset of the strains described in Montooth et al., 2010. Alternative *D. melanogaster OreR* mtDNA (*ore*) and *D. simulans simw^501^* mtDNA are placed on two inbred, wild-type *D. melanogaster* nuclear backgrounds, *OreR* and *AutW132*, using balancer chromosome extraction. The transgenic rescue strains are described in Meiklejohn et al., 2013. In brief, the *Aatm^Ore^*, *Aatm^Aut^* and *Aatm^Ore_V275A^* transgenic constructs containing the complete *Aatm* coding sequence were inserted into the same genomic location using ΦC31-mediated integration. The incompatible *Aatm^Ore^* allele differs from the *Aatm^Ore_V275A^* rescue allele by the A275V SNP in the synthetase coding sequence, and the sequence-verified *Aatm^Aut^* and *Aatm^Ore^* alleles differ by an additional single synonymous SNP that was not manipulated. Stocks homozygous for the transgenes were established, and carry a deficiency spanning the endogenous location of the *Aatm* allele on the second chromosome, balanced over *Cyo*. *Cyo* contains a wild-type copy of the *Aatm* allele matching the *D.mel* reference sequence. Transgenic stocks are crossed to virgin female (*simw^501^*); *OreR* to generate a series of experimental and transgenic control strains (supplementary material Fig. S1). The genetic notation used throughout is: (*mito*); *nuclear*, with the nuclear being the full homozygous chromosome sets for the introgression strains or the inserted *Aatm* allele for the transgenic strains. All fly stocks were kept in a 25°C temperature-controlled incubator with a 12 h on/off light cycle. Flies were density controlled and raised on standard media (2% yeast, 5.2% cornmeal, 11% sugar, 1% tegosept, 0.8% agar). Unless otherwise noted flies used in assays were mated females 15 days post-eclosion.

### Bristle length

Posterior scutellar macrochaetae were measured using a Nikon dissecting microscope at 50× power (10× ocular, 5× zoom objective) with a micrometer scale built into the ocular lens. Flies were frozen at −20°C and bristles were measured from the tip to the base, with flies placed on a bed of cotton so that the shaft of the bristle could be aligned to be parallel with the focal plane of the microscope, allowing standardized measurements to be obtained. 15 flies for each genotype were measured in two blocks from separate genotype builds.

### Flight assay

To test overall flight performance a cylindrical flight tester was used as originally described by Benzer (1973). A 500 ml graduated cylinder was coated on the inside with paraffin oil and flies were gently released through an affixed funnel at the top. Flies will fly immediately outwards and stick to the oil as they strike the walls of the cylinder. Flies with poor flight ability fall further before striking the wall. The cylinder was divided into 10 height bins, and the number of flies stuck at each height was quantified and plotted. 15-day-old female flies were used in this assay; *n*>300.

### Climbing assay

Climbing ability (negative geotaxis) was measured in a custom-engineered lab apparatus. 10-ml plastic pipettes were used as cylindrical tubes (30 cm height×1 cm diameter) and were affixed to a stable backboard. Five flies were placed into a tube and the bottom was then stoppered with foam plugs. These specifications minimize crowding while discouraging flight. The backboard was tapped onto a hard surface applying universal force to knock the flies to the base of the tubes. The apparatus is stood upright and flies are given 30 s to climb, at which time digital photos were taken and the height climbed was measured for each individual fly. The apparatus is rested horizontally and flies are given 15 min of recovery time before being tested again. 15-day-old female flies were used in this assay; *n*>300.

### Mitochondrial morphology and TEM

Indirect flight muscles were dissected from 15-day-old female flies into cold Schneider's Drosophila media, and fixed in a 2.5% Glutaraldehyde/2% Paraformaldehyde solution containing 0.1 M NaCaCo and 0.05 M sucrose. Samples were post-fixed in osmium tetroxide, washed and dehydrated in an ethanol series. Samples were embedded in low viscosity Spurr's resin. Samples were cut on a Reichert Ultracut E microtome in 90-nm sections. A Philips 410 transmission electron microscope was used for imaging.

### Mitochondrial isolation

30 flies were sorted by sex on light CO_2_ and were given at least 48 h recovery time prior to homogenization. Flies were gently homogenized in 1 ml chilled isolation buffer (225 mM mannitol, 75 mM sucrose, 10 mM MOPS, 1 mM EGTA, 0.5% fatty-acid-free BSA, pH 7.2) using a glass-teflon dounce homogenizer. The extracts were centrifuged at 300 ***g*** for 5 min at 4°C. The obtained supernatant was then centrifuged at 6000 ***g*** for 10 min at 4°C to obtain a mitochondrially enriched pellet. The pellet was resuspended in 100 μl respiration buffer (225 mM mannitol, 75 mM sucrose, 10 mM KCl, 10 mM Tris-HCl, 5 mM KH_2_PO_4_, pH 7.2). Freshly prepared mitochondrial isolations were used for respiration or aliquoted and frozen at −80°C for the mitochondrial enzyme complex activity assays. Quantification of mitochondrial protein obtained from the isolation was determined using the BCA protein assay (Thermo Scientific, Rockford, IL, USA).

### Mitochondrial respiration

Respiration rates were determined by oxygen consumption using a Clark-type electrode and metabolic chamber (Hansatech Instruments, Norfolk, UK). A combination of NADH-linked substrate, 5 µmol of pyruvate plus 5 µmol malate, was added to an isolated mitochondrial suspension in 1 ml or respiration buffer held in the respiration chamber at 30°C. 125 nmol ADP was added to generate state III respiration, representative of the maximal ability to generate ATP. State IV, or basal, respiration rates were measured. The ratio of state III to state IV was calculated to define the respiratory control ratio (RCR). Ratios of >3 were considered functionally acceptable for fresh mitochondria preparations (Schriner et al., 2009). Uncoupled respiration rates, indicative of maximal rate of O_2_ consumption, were generated by the addition of 0.5 nmol of FCCP [carbonyl cyanide 4-(trifluoro-methoxy) phenylhydrazone], a chemical uncoupler.

### Blue-native polyacrylamide gel electrophoresis (BN-PAGE)

To obtain mitochondrial complexes in the native state, mitochondrial preps were carried out as described above and the mitochondrial pellets were resuspended in 100 µl 1× Native Sample Buffer (Invitrogen, NY, USA) containing 1% digitonin and 1× protease inhibitors (Roche, CA, USA). Samples were incubated for 15 min on ice, and were centrifuged at 16.1 ***g*** at 4°C for 40 min. Supernatant was aliquoted and frozen at −80°C. Protein was quantified using the BCA Protein Assay (Thermo Scientific, Rockford, IL, USA). 15 μg of mitochondrial protein were run on 3-12% Bis-Tris Native PAGE gels per manual specifications (Novex, Life Technologies, NY, USA). 1× Native PAGE Running buffer and 1× Dark Blue cathode buffer were used (Invitrogen) for electrophoresis. NativeMark Protein standard (Invitrogen) was used as the molecular weight marker. ImageJ software was used to quantify band intensity. Band intensity was normalized to internal BSA band present in samples (BSA added during mitochondrial isolation procedure). In images, full lanes were moved from original gel locations for genotype consistency, no manipulations were made within lanes.

### Mitochondrial complex activity assays

Mitochondrial isolations were performed as described above. Enzyme activity assays were performed as previously described (Meiklejohn et al., 2013). In brief, protein quantification was used to standardize the amount of protein added to each reaction. The specific activity of Complex I was determined following the oxidation of NADH at 340 nm with the coenzyme Q analog decylubiquinone as the electron acceptor. The catalytic activity of Complex II was monitored by the reduction of DCPIP at 600 nm, and was inhibited with 400 mM malonate. Complex III activity was measured by monitoring the reduction of cytochrome *c* at 550 nm, and was inhibited with 5 µg/ml antimycin A. Complex IV (cytochrome *c* oxidase) activity was measured by determining the rate of oxidation of reduced cytochrome *c* at 550 nm, and was inhibited with 4 mM KCN. To measure citrate synthase activity, the rate-limiting reaction of citrate synthase was coupled to a chemical reaction in which DTNB reacts with CoA-SH and the absorbance of the product is measured at 412 nm. Specific reaction mixtures are described in Meiklejohn et al., 2013.

### ^35^S-methionine incorporation

*De novo* translation was measured by incorporation of ^35^S-methionine into synthesized proteins. Female flies were placed on standard media supplemented with 100 µCi/ml ^35^S-methionine for 48 h; *n*>4, 20 flies per *n*. Mitochondrial preparations were carried out as described above (using a BSA free isolation buffer: 210 mM Mannitol, 70 mM Sucrose, 5 mM HEPES, 1 mM EDTA, pH 7.4, 1× protease inhibitor), and the supernatant from the low speed spin was taken as the cytosolic fraction. 1% triton was added to fractions and proteins were precipitated in 10% trichloroacetic acid. A Beckman LS 6500 scintillation counter was used to measure radiolabeled methionine incorporation. Values were normalized to protein content in each fraction (BCA method).

### Statistical analyses

Analysis of variance models were used in conjunction with Tukey's post-hoc contrasts to compare the phenotypic effects of the transgenic alleles. For non-normally distributed data sets (climbing and flight), Kruskal–Wallis one-way analysis of variance were used in conjunction with Wilcoxon rank sum tests. Bonferroni-corrected *P*-values are provided to account for multiple testing. Analyses were conducted using the statistical package R version 2.15.0.

## Supplementary Material

Supplementary Material
