# Supplementary material for: A Drosophila model for mito-nuclear diseases generated by an incompatible interaction between tRNA and tRNA synthetase
Source: Dis Model Mech. 2015 Aug 1;8(8):843–54. doi: 10.1242/dmm.019323 (PMC4527286; doi:10.1242/dmm.019323)
Supplement: Supplementary Material [file supp_8_8_843__index.html]

Supplementary Material 

# A *Drosophila* model for mito-nuclear diseases generated by an incompatible interaction between tRNA and tRNA synthetase

## DMM019323 Supplementary Material

- Supplementary Material
