# Supplementary material for: A Drosophila model for mito-nuclear diseases generated by an incompatible interaction between tRNA and tRNA synthetase
Source: Dis Model Mech. 2015 Aug 1;8(8):843–54. doi: 10.1242/dmm.019323 (PMC4527286; doi:10.1242/dmm.019323)
Supplement: Supplementary Material [file supp_019323_DMM019323supp.pdf]

Supplementary Tables and Figures.

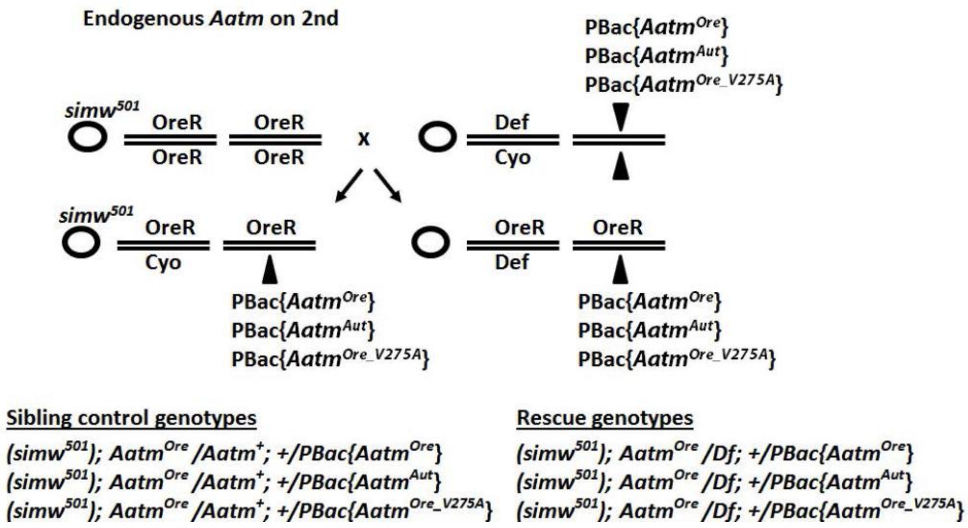

Figure S1. Crossing scheme for the generation of the Aatm rescue genotypes, and their sibling controls. OreR refers to the wild type chromosome, Def refers to a deficiency spanning the native Aatm locus, CyO refers to the balancer chromosome for the Drosophila 2nd chromosome, PBac{$Aatm^{Ore}$} refers to the transgenic construct inserted in to the 3rd chromosome at the point of the triangle. The rescue genotypes noted as (sim^{w501});$Aatm^{Ore}$/Df;+/PBac{$Aatm^{Ore}$} have sim^{w501} mtDNA; the OreR allele of Aatm over the deficiency; a wild (+) 3rd chromosome over the transgenic PBac allele. The Ore allele of Aatm is mutant (Valine), and the Aut allele is wild type (Alanine). The rescue construct replaces the Valine with the Alanine in an Ore Aatm allele. The sibling control genotypes have a wild type Aatm allele on the second chromosome, so carry a normal function for the mt-$tRNA^{Tyr}$. See Meiklejohn et al. 2013 for details of genotype construction.

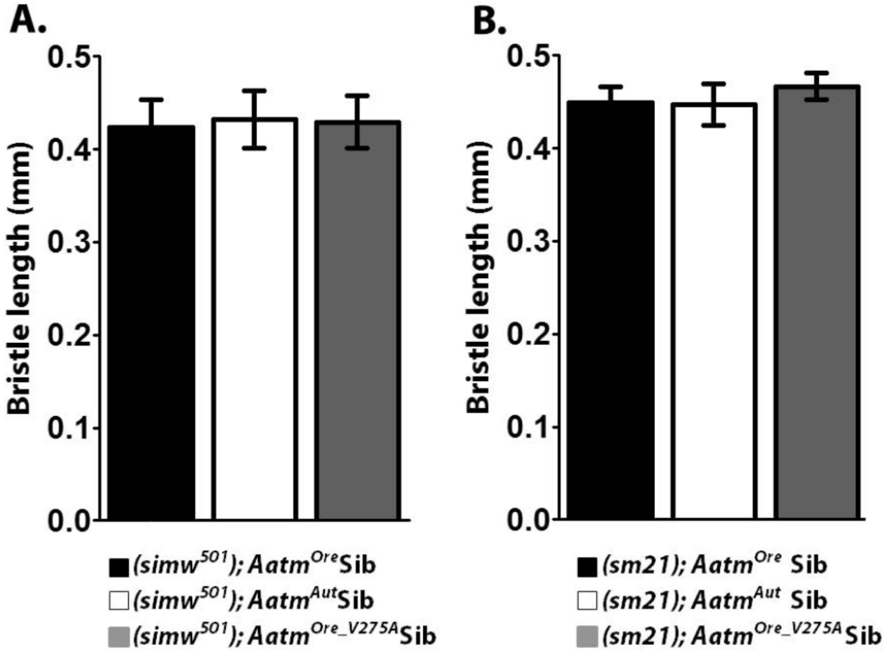

Figure S2. Bristle length in sibling control genotypes carrying a simw510 or a sm21 mtDNA. There is no significant difference among all genotypes in bristle length.

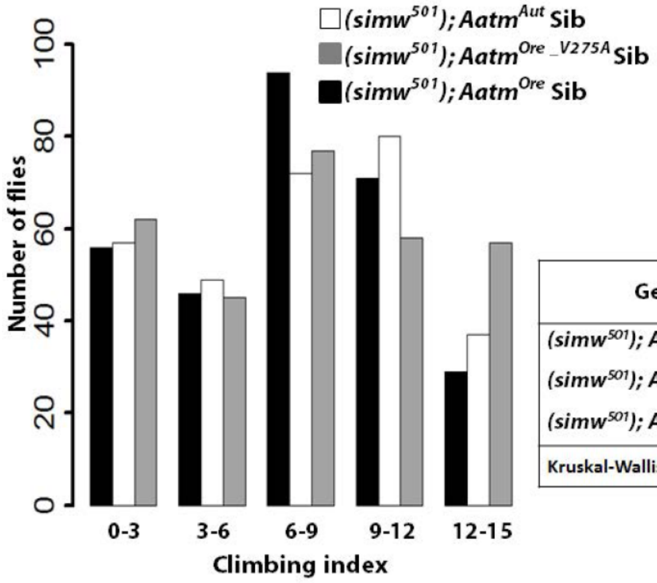

Genotype	Mean height score ± sem
(simw^{501}); Aatm^{Ore} **Sib**	7.45 ± 0.23[a]
(simw^{501}); Aatm^{Aut} **Sib**	7.71 ± 0.24[a]
(simw^{501}); Aatm^{Ore_V275A} **Sib**	7.75 ± 0.26[a]
Kruskal-Wallis chi-squared = 1.41 df = 2, *P* = 0.49	

Figure S3. Climbing data for the sibling control genotypes. There is no significant difference among the three genotypes in climbing index.

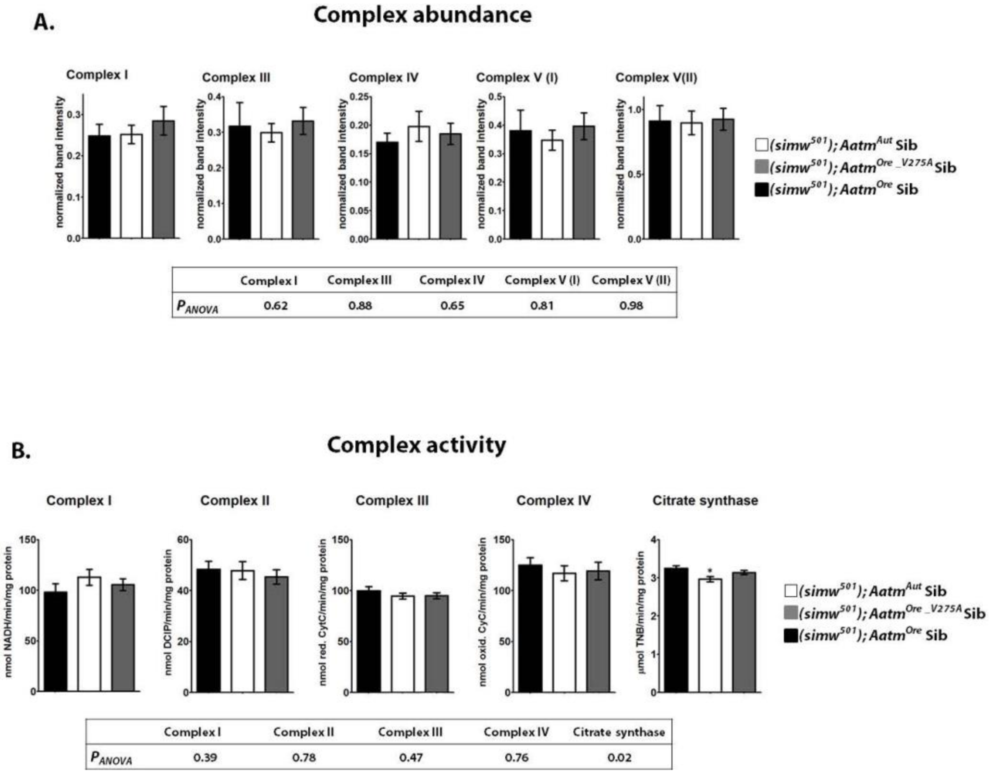

	Complex I	Complex III	Complex IV	Complex V (I)	Complex V (II)
P_{ANOVA}	0.62	0.88	0.65	0.81	0.98

	Complex I	Complex II	Complex III	Complex IV	Citrate synthase
P_{ANOVA}	0.39	0.78	0.47	0.76	0.02

Figure S4. A: abundance of mitochondrial OXPHOS complexes for the sibling control genotypes from blue native PAGE. There is no significant difference among the three genotypes for any of the complexes. B: Enzymatic activity of the four electron transport chain complexes and citrate synthase among the sibling control genotypes. There is no significant variation in enzymatic activity among the genotypes.

Figure S5

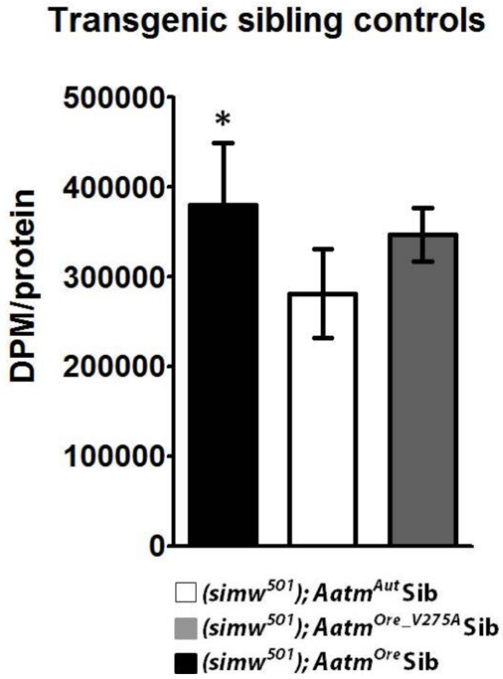

Figure S6

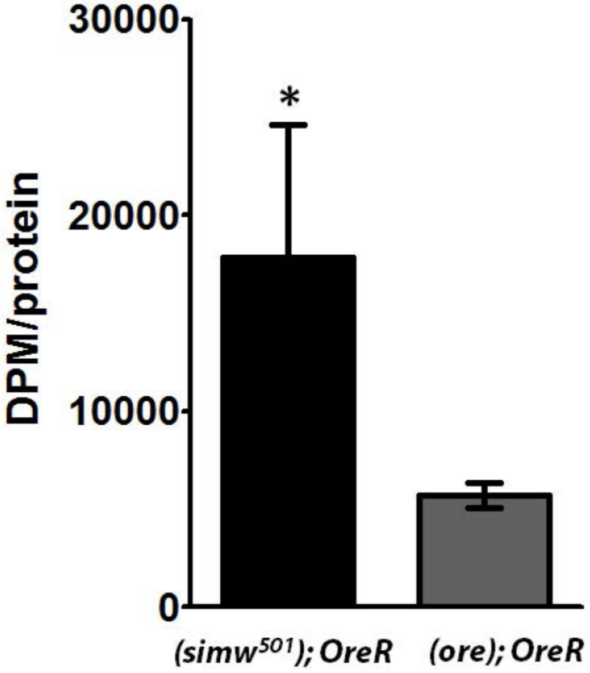

Figure S5 and S6. Estimation of translation initiation from ^{35}S-methionine labeling of mitochondrial fractions among the sibling control genotypes (S5) and original introgression strains (S6). The incompatible pairings of mt-tRNA and nuclear Aatm allele show elevated label incorporation suggesting compensatory mechanisms to increase protein translation in mitochondria.

Table S1. Illustration of the mutations in the mtDNA-tRNATyr gene and nuclear encoded mitochondrial tRNATyr sythetase. The star symbol denotes the presence of a mutation in the respective gene (two stars if diploid), and the lack of a symbol denotes a wild type allele. The triangle symbol denotes the presence of a wild type Aatm allele provided by the transgenic construct (two triangles if diploid).

Mito-nuclear genotypes	mtDNA tRNA mutation	Nuclear synthetase mutation
(simw^{501}); OreR	✦	✦ ✦
(ore); OreR		✦ ✦
(simw^{501}); Aut	✦	
(ore); Aut		

✦ Mutation

▼ Compatible transgenic insert

Transgenic Aatm Strains	mtDNA tRNA mutation	Nuclear synthetase mutation	Nuclear compatible *Aatm* allele	
**Sibling controls genotypes**				
(simw^{501}); Aatm^{Ore} Sib	✦	✦ ✦	▼	provided by *Cyo* balancer
(simw^{501}); Aatm^{Aut} Sib	✦	✦	▼ ▼	provided by *Cyo* balancer, transgenic *Aatm^{Aut}* allele
(simw^{501}); Aatm^{Ore_V275A} Sib	✦	✦	▼ ▼	provided by *Cyo* balancer, transgenic *Aatm^{Ore_V275A}* rescue allele
**Rescue genotypes**				
(simw^{501}); Aatm^{Ore}	✦	✦ ✦		
(simw^{501}); Aatm^{Aut}	✦	✦	▼	transgenic *Aatm^{Aut}* allele
(simw^{501}); Aatm^{Ore_V275A}	✦	✦	▼	transgenic *Aatm^{Ore_V275A}* rescue allele

Table S2. Mitochondrial oxygen consumption rates and p-values for the three sibling control genotypes. State III is ADP dependent, State IV is the background or leakage rate, RCR is state III / State IV, and Uncoupled is maximal rate after addition of the uncoupler FCCP. There is no significant variation among the genotypes for any of these respiration rates.

Genotype	State III	State IV	RCR	Uncoupled
(simw^{501}); Aatm^{Ore} Sib	10.3 ± 0.24^{a}	1.85 ± 0.06^{a}	6.2 ± 0.34^{a}	18.1 ± 0.29^{a}
(simw^{501}); Aatm^{Aut} Sib	10.2 ± 0.24^{a}	1.77 ± 0.05^{a}	6.2 ± 0.25^{a}	17.7 ± 0.25^{a}
(simw^{501}); Aatm^{Ore_V275A} Sib	10.1 ± 0.19^{a}	1.93 ± 0.06^{a}	5.7 ± 0.26^{a}	18.1 ± 0.27^{a}
P_{ANOVA}	0.93	0.58	0.73	0.85

Table S3. Tyrosine composition of the 13 mtDNA-encoded subunits of OXPHOS complexes. The proportion is relatively similar across genes, but the total number of tyrosine residues is greatest in complex I, which has the most subunits and longer genes. This is consistent with the evidence for a larger impact of the tRNA mutation on complex I activity.

Complex	Dmel	Amino acids	Number of tyrosines	% Tyrosine
Complex III	Cyt b	378	19	0.05
	COX1	511	18	0.04
	COX2	228	9	0.04
	COX3	262	12	0.05
Complex IV	COX	1001	39	0.04
	ND1	312	19	0.06
	ND2	341	11	0.03
	ND3	117	2	0.02
	ND4	446	25	0.06
	ND4L	96	5	0.05
	ND5	574	29	0.05
	ND6	174	7	0.04
Complex I	ND	2060	98	0.05
	ATP6	224	7	0.03
	ATP8	53	3	0.06
Complex V	ATP	277	10	0.04

Table S4. Pairwise contrasts between genotypes for flight ability. P-values are listed for Wilcoxon rank sum tests on flight index values, corrected for multiple tests.

Pairwise contrasts for flight data

Wilcoxon rank sum test with continuity correction, Bonferoni corrected p-values

Contrasts	*P*
**Mito-nuclear strains**	
(*simw*^{501}); *OreR* - (ore); *Ore*	0.0043
(*simw*^{501}); *OreR* - (*simw*^{501}); *Aut*	1.3e-15
(*simw*^{501}); *OreR* - (ore); *Aut*	2.7e-12
(ore); *Ore* - (*simw*^{501}); *Aut*	1.3e-8
(ore); *Ore* - (ore); *Aut*	0.0011
(*simw*^{501}); *Aut* - (ore); *Aut*	0.33
**Transgenic rescue strains**	
(*simw*^{501}); *Aatm*^{Ore} - (*simw*^{501}); *Aatm*^{Aut}	0.00083
(*simw*^{501}); *Aatm*^{Ore} - (*simw*^{501}); *Aatm*$^{Ore\_V275A}$	0.033
(*simw*^{501}); *Aatm*^{Aut} - (*simw*^{501}); *Aatm*$^{Ore\_V275A}$	0.77

Pairwise contrasts for climbing data

Wilcoxon rank sum test with continuity correction, Bonferoni corrected p-values

Contrasts	*P*
**Mito-nuclear stains**	
(*simw*^{501}); *OreR* - (ore); *Ore*	0.11
(*simw*^{501}); *OreR* - (*simw*^{501}); *Aut*	1.3e-15
(*simw*^{501}); *OreR* - (ore); *Aut*	1.3e-15
(ore); *Ore* - (*simw*^{501}); *Aut*	1.3e-15
(ore); *Ore* - (ore); *Aut*	1.3e-15
(*simw*^{501}); *Aut* - (ore); *Aut*	0.0055
**Transgenic rescue strains**	
(*simw*^{501}); *Aatm*^{Ore} - (*simw*^{501}); *Aatm*^{Aut}	3.8e-6
(*simw*^{501}); *Aatm*^{Ore} - (*simw*^{501}); *Aatm*$^{Ore\_V275A}$	0.0034
(*simw*^{501}); *Aatm*^{Aut} - (*simw*^{501}); *Aatm*$^{Ore\_V275A}$	0.041
**Transgenic sibling controls**	
(*simw*^{501}); *Aatm*^{Ore} *Sib* - (*simw*^{501}); *Aatm*^{Aut} *Sib*	0.38
(*simw*^{501}); *Aatm*^{Ore} *Sib*- (*simw*^{501}); *Aatm*$^{Ore\_V275A}$ *Sib*	0.27
(*simw*^{501}); *Aatm*^{Aut} *Sib* - (*simw*^{501}); *Aatm*$^{Ore\_V275A}$ *Sib*	0.74

Table S5. Pairwise contrasts for mitochondrial morphology from TEM images. P-values shown are from one-way ANOVA using Tukey HSD correction for all pairwise comparisons.

Pairwise contrasts for mitochondrial morphology TEM data

Tukey HSD

**Contrasts**	***P*** **Mitochondrial size**	***P*** **Empty space**	***P*** **Mitochondria/ unit area**
**Mito-nuclear strains**			
($simw^{501}$); *OreR* - (ore); *Ore*	1.2e-3	0.18	0.031
($simw^{501}$); *OreR* - ($simw^{501}$); *Aut*	0.91	0 094	
($simw^{501}$); *OreR* - (ore); *Aut*	0.28	0.20	0.82
(ore); *Ore* - ($simw^{501}$); *Aut*	0.08	1.0e-3	0.057
(ore); *Ore* - (ore); *Aut*	2.6e-6	1.4e-3	0.10
($simw^{501}$); *Aut* - (ore); *Aut*	0.17	0.89	0.97
**Transgenic rescue strains**			
($simw^{501}$); $Aatm^{Ore}$ - ($simw^{501}$); $Aatm^{Aut}$	0.016	2.2e-3	
($simw^{501}$); $Aatm^{Ore}$ - ($simw^{501}$); $Aatm^{Ore\_V275A}$	1.2e-4	0.057	0.011
($simw^{501}$); $Aatm^{Aut}$ - ($simw^{501}$); $Aatm^{Ore\_V275A}$	0.43		

Table S6. Pairwise contrasts for mitochondrial respiration data. P-values shown are from one-way ANOVA using Tukey HSD correction for all pairwise comparisons.

Pairwise contrasts for mitochondrial respiration data

Tukey HSD

Contrasts	*P* State III	*P* State IV	*P* RCR	*P* Uncoupled
**Mito-nuclear strains**				
(*simw*^{501}); *OreR* - (ore); *Ore*	9e-3	0.36	0.63	0.014
(*simw*^{501}); *OreR* - (*simw*^{501}); *Aut*	6e-6	0.97	0.019	7.3e-4
(*simw*^{501}); *OreR* - (ore); *Aut*	2e-7	0.56	0.014	1.6e-6
(ore); *Ore* - (*simw*^{501}); *Aut*	0.090	0.70	0.25	0.74
(ore); *Ore* - (ore); *Aut*	0.011	0.99	0.23	0.068
(*simw*^{501}); *Aut* - (ore); *Aut*	0.90	0.86	1.0	0.51
**Transgenic rescue strains**				
(*simw*^{501}); *Aatm*^{Ore} - (*simw*^{501}); *Aatm*^{Aut}	1.0	0.10	0.15	0.05
(*simw*^{501}); *Aatm*^{Ore} - (*simw*^{501}); *Aatm*$^{Ore\_V275A}$	0.30	0.62	0.98	0.88
(*simw*^{501}); *Aatm*^{Aut} - (*simw*^{501}); *Aatm*$^{Ore\_V275A}$	0.30	0.49	0.10	0.14
**Transgenic sibling controls**				
(*simw*^{501}); *Aatm*^{Ore} *Sib* - (*simw*^{501}); *Aatm*^{Aut} *Sib*	0.96	0.86	0.99	1.0
(*simw*^{501}); *Aatm*^{Ore} *Sib*- (*simw*^{501}); *Aatm*$^{Ore\_V275A}$ *Sib*	0.93	0.87	0.74	0.87
(*simw*^{501}); *Aatm*^{Aut} *Sib* - (*simw*^{501}); *Aatm*$^{Ore\_V275A}$ *Sib*	0.99	0.55	0.81	0.88

Table S7. Pairwise contrasts for mitochondrial complex quantification from BN-PAGE (top) and enzyme activity (bottom). P-values shown are from one-way ANOVA using Tukey HSD correction for all pairwise comparisons.

Pairwise contrasts for BN-PAGE gels quantification and mitochondrial complex activity data

BN-PAGE Tukey HSD Contrasts	*P* Complex I	*P* Complex III	*P* Complex IV	*P* Complex V(I)	*P* Complex V(II)
**Transgenic rescue strains**					
(simw^{501}); Aatm^{Ore} - (simw^{501}); Aatm^{Aut}	8.0e-4	0.044	0.53	4.5e-3	0.23
(simw^{501}); Aatm^{Ore} - (simw^{501}); Aatm^{Ore_V275A}	0.016	1.0	0.45	0.040	0.61
(simw^{501}); Aatm^{Aut} - (simw^{501}); Aatm^{Ore_V275A}	0.38	0.044	0.99	0.57	0.74
**Transgenic sibling controls**					
(simw^{501}); Aatm^{Ore} Sib - (simw^{501}); Aatm^{Aut} Sib	1.0	0.96	0.87	0.91	1.0
(simw^{501}); Aatm^{Ore} Sib- (simw^{501}); Aatm^{Ore_V275A} Sib	0.65	0.97	0.62	0.98	1.00
(simw^{501}); Aatm^{Aut} Sib - (simw^{501}); Aatm^{Ore_V275A} Sib	0.71	0.87	0.90	0.80	0.98

Complex activity Tukey HSD Contrasts	*P* Complex I	*P* Complex II	*P* Complex III	*P* Complex IV	*P* Citrate Synthase
**Transgenic rescue strains**					
(simw^{501}); Aatm^{Ore} - (simw^{501}); Aatm^{Aut}	3.2e-5	0.97	0.81	0.016	0.77
(simw^{501}); Aatm^{Ore} - (simw^{501}); Aatm^{Ore_V275A}	0.041	0.97	0.98	0.03	1.0
(simw^{501}); Aatm^{Aut} - (simw^{501}); Aatm^{Ore_V275A}	0.047	0.87	0.91	0.97	0.72
**Transgenic sibling controls**					
(simw^{501}); Aatm^{Ore} Sib - (simw^{501}); Aatm^{Aut} Sib	0.36	0.99	0.52	0.76	0.015
(simw^{501}); Aatm^{Ore} Sib- (simw^{501}); Aatm^{Ore_V275A} Sib	0.76	0.78	0.55	0.86	0.53
(simw^{501}); Aatm^{Aut} Sib - (simw^{501}); Aatm^{Ore_V275A} Sib	0.77	0.84	1.0	0.98	0.18

Table S8. Pairwise contrasts for ^{35}S-methionine incorporation in mitochondrial extracts of different mitonuclear genotypes. P-values shown are from one-way ANOVA using Tukey HSD correction for all pairwise comparisons.

Pairwise contrasts for ^{35}S-methionine incorporation

Tukey HSD

Contrasts	***P*** ^{35}S-methionine incorporation
**Mito-nuclear strains**	
(*simw*^{501}); *OreR* - (ore); *Ore*	0.10
(*simw*^{501}); *OreR* - (*simw*^{501}); *Aut*	1.0
(*simw*^{501}); *OreR* - (ore); *Aut*	0.16
(ore); *Ore* - (*simw*^{501}); *Aut*	0.14
(ore); *Ore* - (ore); *Aut*	2.4e-3
(*simw*^{501}); *Aut* - (ore); *Aut*	0.30
**Transgenic rescue strains**	
(*simw*^{501}); *Aatm*^{Ore} - (*simw*^{501}); *Aatm*^{Aut}	1.1e-6
(*simw*^{501}); *Aatm*^{Ore} - (*simw*^{501}); *Aatm*$^{Ore\_V275A}$	3.9e-6
(*simw*^{501}); *Aatm*^{Aut} - (*simw*^{501}); *Aatm*$^{Ore\_V275A}$	0.9
**Transgenic sibling controls**	
(*simw*^{501}); *Aatm*^{Ore} *Sib* - (*simw*^{501}); *Aatm*^{Aut} *Sib*	0.012
(*simw*^{501}); *Aatm*^{Ore} *Sib*- (*simw*^{501}); *Aatm*$^{Ore\_V275A}$ *Sib*	0.58
(*simw*^{501}); *Aatm*^{Aut} *Sib* - (*simw*^{501}); *Aatm*$^{Ore\_V275A}$ *Sib*	0.12
